# Coping Strategies in Migraine without Aura: A Cross-Sectional Study

**DOI:** 10.1155/2019/5808610

**Published:** 2019-05-05

**Authors:** Antonio Russo, Gabriella Santangelo, Alessandro Tessitore, Marcello Silvestro, Francesca Trojsi, Antonio De Mase, Federica Garramone, Luigi Trojano, Gioacchino Tedeschi

**Affiliations:** ^1^Headache Center, Department of Medical, Surgical, Neurological, Metabolic and Aging Sciences, University of Campania “Luigi Vanvitelli”, Naples 80138, Italy; ^2^Institute for Diagnosis and Care “Hermitage Capodimonte”, Naples 80100, Italy; ^3^Department of Psychology, University of Campania “Luigi Vanvitelli”, Caserta 81100, Italy; ^4^ICS Maugeri, Institute of Telese, IRCCS, Telese Terme, BN 82037, Italy

## Abstract

**Background:**

In the context of a causal relationship between stress and migraine, coping strategies are aimed at managing stressful life events and reducing the distressing emotions connected to them.

**Methods:**

Sixty-one consecutive patients with migraine without aura (MwoA) and sixty-one healthy controls (HCs) completed three self-report questionnaires assessing a broad range of coping (cognitive and behavioural) strategies: the Coping Orientation to Problems Experienced (COPE), the Coping Inventory for Stressful Situation (CISS), and the Proactive Coping Inventory (PCI). Moreover, the Perceived Stress Scale (PSS), a scale measuring self-perception of stress, global cognitive functioning, depressive symptoms, apathy, state, and trait anxiety, was administered to all participants.

**Results:**

No significant difference was found on the scales and subscales of PCI and CISS as well as in the PSS between MwoA patients and HCs. However, the two groups showed different scores in the subscale “turning to religion” of COPE (22.08 ± 5.19 in migraineurs vs. 24.70 ± 4.44 in HCs, *p* = 0.003). A significant negative correlation of the turning to religion score with the HIT-6 score was found.

**Conclusions:**

The present study revealed that MwoA patients show a significantly reduced use of the “turning to religion” approach, an emotion-focused coping strategy. Although migraine patients appeared to be less oriented to transcendent (that means a reduced utilization of an adaptive coping strategy), they did not perceive daily living as more stressful than HCs. Finally, the reduced utilization of the “turning to religion” coping strategy is associated with a great impact of migraine on ability to function on the job or at school, at home, and in social situations in migraine patients.

## 1. Introduction

Migraine is a common primary headache due to a genetic predisposition of episodic activation and sensitization of the trigeminovascular pain pathway [1]. Although migraine pathophysiology is still a matter of debate, it has been argued that both biological predisposition and conditioning may trigger a migraine attack [2]. Among trigger factors, the critical role of stressors has been recently underlined [3].

Coping is defined as the process of executing a response to a stressor, where stress is viewed as the experience of encountering relevant difficulties in one's goal-related efforts [4] and exists when an individual perceives the achievement of a desired goal as impossible or detects possible future punishments [5]. Thus, coping has been described as an individual's attempt to use cognitive and behavioural strategies to manage and regulate pressures, demands, and emotions in response to stress [6].

In the last years, a relationship between recurrence of migraine episodes and maladaptive coping styles has been suggested, but this issue has been poorly investigated. One study showed that migraineurs tend to seek social support less than nonmigraineurs; moreover, they are more submissive and optimistic in coping with problems than nonmigraineurs [7]. Two further studies evaluated emotional, cognitive, and coping reactions to pain experience in migraine without aura (MwoA), in tension-type headache (TTH), and in chronic migraine (CM) [8, 9]. Migraine sufferers were characterized by pronounced psychological abnormalities during the headache phase, demonstrating a larger use of maladaptive coping behaviour in migraineurs than in patients with TTH [8]. Other observations suggested that migraine patients are prone to use internally focused coping strategies such as nonverbal complaints, suppressive thoughts, and decreased search for social support suggesting a contracted attitude toward affective support coming from other people [8, 28].

Another study showed that the persistency to maladaptive pain coping strategies was a risk factor for recurrent relapses in chronic migraine [9]. Taken together, these studies revealed an association between maladaptive strategies and migraine, but are characterized by some limitations: the inclusion of patients heterogeneous for migraine phenotype, the absence of a comparison with healthy subjects, the inclusion of migraineurs and nonmigraineurs not matched for age, and the use of questionnaires to assess coping strategies specific for pain. It is known that many different types of coping strategies (i.e., problem-focused, emotion-focused, and avoidance-oriented) can be employed to cope the stressful events; however, until now, few studies have systematically investigated the relationship between a specific type of migraine and many different coping responses. Therefore, we performed a study to explore problem-focused and emotion-focused strategies [6] and avoidance-oriented strategies in patients with migraine without aura [10] compared to age- and sex-matched healthy controls (HCs). To achieve this aim, we decided to use two different questionnaires, the Coping Orientation to Problems Experienced (COPE) [11] and Coping Inventory for Stressful Situation (CISS) [12], assessing basic coping strategies by means of the evaluation of different coping responses. Moreover, for the first time, we used the Proactive Coping Inventory (PCI) [13] in MwoA patients to evaluate the proactive coping defined as consisting of “effort to develop general resources, thereby facilitating the achievement of personal goals and working toward personal growth” [14]. Moreover, to investigate the relationship between coping strategies employed by migraineurs and the degree to which they perceive life events as stressful, the Perceived Stress Scale (PSS) has been administered [15]. Finally, we investigated if specific coping strategies employed by migraineurs are related to depressive symptoms, trait, or state anxiety, an aspect not explored in the previous studies.

All patients were both drug-naïve for preventive migraine therapies and studied during an interictal period to avoid confounds associated with pharmacological and migraine attack's effects.

Taking into account the previous studies on a larger use of maladaptive pain coping strategies in migraineurs against pain, we hypothesized that MwoA patients might adopt maladaptive coping strategies as compared to HCs.

## 2. Materials and Methods

In the present study, 61 consecutive outpatients with MwoA referred to the Headache Center of the University of Campania “Luigi Vanvitelli” were screened from March 2015 to March 2016. Inclusion criteria were (1) MwoA according to International Headache Society Diagnostic Criteria [11], (2) normal neurological evaluation, (3) absence of other headache, psychiatric, or somatic disorders, (4) no cognitive decline (defined by an age and education adjusted total score on Italian version of the Montreal Cognitive Assessment (MoCA) < 15.5) [16], and (5) lack of present or past therapy with antimigraine agents.

We also enrolled a sample of 61 healthy controls (HCs) meeting the same selection criteria as above with the exclusion of MwoA and matched for sex, age (both groups were in a range between 23 and 45 years old), and education with patients. HCs were recruited from among patients' friends, employees at the clinic or university centres, and were included if they met the following selection criteria: lack of history of migraine or any other type of headache and/or current diagnosis of migraine according to clinical criteria, lack of history of or actual psychiatric diseases (e.g., major depression or psychosis according to DSM-V criteria), and no use of psychoactive drugs.

The clinical aspects of migraineurs such as disease duration, migraine attacks per month, and mean pain intensity during migraine attacks (evaluated by means of numerical rating scale (NRS)) were recorded. Patients' headache-related disability and the migraine impact on patients' life were obtained using Migraine Disability Assessment Scale [17] (MIDAS) and the Headache Impact Test-6 (HIT-6), respectively [18].

Patients and HCs completed three self-report questionnaires assessing a broad range of coping (cognitive and behavioural) strategies employed to cope the general stressful events: the COPE, the PCI, and the CISS [11–13, 14].

The COPE is a 60-item self-report measure of strategies used by individuals to cope with problems and stress, both adaptive and maladaptive [11]. Each item can be answered on a four-point Likert-type scale ranging from “I usually don't do this at all” to “I usually do this a lot.” The tool gives a score on the following coping approaches: positive reframing, social support, acceptance, denial, and turning to religion.

The PCI is a 55-item self-report, to be answered on a four-point Likert-type scale ranging from “not at all true” to “completely true.” The PCI consists of seven subscales: the proactive coping scale, the preventive coping scale, the reflective coping scale, the strategic planning scale, the instrumental support seeking scale, the emotional support seeking scale, and the avoidance coping scale [13]. These subscales measure different dimensions of a proactive approach to coping. Greenglass reported good psychometric properties of the instrument, including acceptable internal consistency and validity [14].

The CISS is a 48-item self-measure assessing five basic coping strategies: task solution, rumination, aggression, distraction, and social diversion [12]. Each item can be answered on a five-point Likert-type scale ranging from “not at all” to “very much”.

Last, all participants completed the PSS, a scale measuring self-perception of stress during the last month [15]. It is a measure of the degree to which life events are appraised as stressful. It consists of 10 items; each item can be answered on a four-point Likert-type scale ranging from “not at all” to “very much”.

To assess global cognitive functioning, all MwoA patients and HCs underwent the Italian version of Montreal Cognitive Assessment (MoCA) [16], a tool which evaluate several cognitive domains, memory, attention, language, and orientation, and visuospatial and executive function domains. The MoCA total score ranges from 0 to 30, and a value of age- and education-adjusted total MoCA score lower than 15.5 is suggestive for the presence of cognitive decline. As for the psychological profile, all MwoA patients and HCs underwent the Beck Depression Inventory II (BDI-II) [19], self-version of Apathy Evaluation Scale (AES-S) [20], and the State and Trait Anxiety Inventory 1 and 2 (STAI-Y-1 and 2) [21].

The BDI-II is a questionnaire consisting of 21 items, designed to assess severity of depressive symptoms. The total score ranges from 0 to 63, with higher scores reflecting higher levels of depression.

Apathy was evaluated by the Apathy Evaluation Scale, assessing four apathy domains: behavioural,” “emotional,” “cognitive,” and “other” (i.e., reduction or loss of motivation, initiative, and accurate understanding of one's problems). The total score ranges from 18 to 72. Clinically significant apathy was identified according to a cut-off score of 38.

Severity of anxiety was assessed by means of the State-Trait Anxiety Inventory (STAI). STAI subscores can differentiate between the temporary condition of state anxiety (i.e., the temporary fear and nervousness due to a certain situation) and the more general and long-lasting trait anxiety (i.e., the stress, worry, and discomfort perceived in typical situations on a daily basis). We recorded information about religious beliefs of all participants (both MwoA patients and HCs).

All demographic and clinical data are shown in Table 1.

### 2.1. Standard Protocol Approvals, Registrations, and Patient Consents

All participants gave their written informed consent. The study was performed in accordance with the ethical standards laid down in the 1964 Declaration of Helsinki and was approved by the local ethics committee.

### 2.2. Statistical Analysis

The two groups (MwoA patients and HCs) were compared on continuous variables by independent *t*-tests. Bonferroni correction was applied (0.05/17 = 0.003). In the MwoA group, correlational analysis were performed between behavioural variables (BDI-II and STAI-Y-1 and 2), clinical variables, MoCA score, Perceived Stress Scale (PSS), and the coping strategies where *t*-tests revealed a significant difference between MwoA and HC groups. The association was assessed by Pearson's coefficient. Statistical analyses were performed with the SPSS software (version 19; SPSS Inc., Chicago, IL, USA).

## 3. Results

In the present study, 61 MwoA patients and 61 HCs were included. Significant difference between MwoA patients and HCs was found in global cognitive functioning, but not on depressive symptoms, state, and trait anxiety. Similarly, no significant difference was found on the scales and subscales of PCI and CISS as well as in the PSS. The two groups showed different scores in the subscale “turning to religion” of COPE (22.08 ± 5.19 in MwoA patients vs. 24.70 ± 4.44 in HCs, *p* = 0.003). Both MwoA patients and HCs referred a Catholic (e.g., Roman Catholic church) background. In this frame, no subject showed affinity to extremist religious beliefs or groups. All results are shown in Table 2.

### 3.1. Correlation Analysis

In the MwoA group, after Bonferroni correction (0.05/5 = 0.01) no significant correlation of the turning to religion score was found with STAI-Y-2 (*r* = 0.274, *p* = 0.032), STAI-Y-1 (*r* = 0.245, *p* = 0.057), BDI-II (*r* = 0.052, *p* = 0.693), PSS (*r* = 0.099, *p* = 0.449), and MoCA scores (*r* = −0.164, *p* = 0.231). Moreover, a significant but weak, negative correlation of the turning to religion score with the HIT-6 score was found (*r* = −0.276, *p* = 0.039). After Bonferroni correction (0.05/5 = 0.01), this correlation is not significant.

## 4. Discussion

The present study revealed that MwoA patients show no abnormalities neither in the different dimensions of basic coping strategies nor in a proactive approach to coping. However, a significantly reduced use of the “turning to religion” approach, an emotion-focused coping strategy [11], has been observed in MwoA patients when compared to HCs. Interestingly, although migraine patients appeared to be less oriented to transcendent (that means a reduced utilization of an adaptive coping strategy in the dynamic interaction with the challenges and stressful events of the life), they did not perceive daily living as more stressful than HCs. Moreover, we found that the reduced use of the “turning to religion” coping strategy is associated with a great impact of migraine on ability to function on the job or at school, at home, and in social situations in migraine patients. However, after Bonferroni correction, this finding became not significant.

Although there is disagreement regarding description of coping, some authors have comprehensively defined coping as “constantly changing cognitive and behavioural efforts to manage specific external and/or internal demands that are appraised as taxing or exceeding the resources of the person” [22]. It is well known that our brain responds to stressful or potentially stressful events by activating neural mediators and modifying behaviours to maintain physiological stability (“allostasis”) [23]. In this frame, the concept of coping refers to both the flexible strategies employed to face stressful situations and the emotions connected to them [6, 22]. Consequently, by behaving and thinking in a specific way, the level of stress could be decreased leading to successful adaptation [24]. However, when behavioural stressors are too frequent or severe, allostatic responses can become dysregulated and maladaptive (so-called “allostatic load”) [23].

In the present study, we did not find any difference between migraineurs and nonmigraineurs on problem-oriented, emotion-oriented, and avoidance-oriented strategies evaluated by two different tools. However, we found that migraine patients have revealed a low tendency to “turning to religion,” and they seem to be less prone to seek an external support than nonmigraineurs. This finding is in keeping with the previous validation studies that, using factorial analysis, demonstrated that turning to religion might form a higher-order factor in itself, since this scale did not load on any other factor [25]. In other terms, turning to religion seems to be a strategy of coping unrelated to the other coping types and migraineurs seem not to rely on this coping response [11].

It is well demonstrated that transcendent meaning, religiosity, and church attendance play a positive role in coping with stressful events catalysing a greater personal empowerment, hope, and strength in the face of adversity [26]. However, we believe that “turning to religion” coping strategy cannot be considered as a mere transcendent orientation and that spirituality is broader than religion [27, 28]. Indeed, according with the previous interpretation, spiritual coping strategies incorporate both the religious and existential methods of coping [29]. Our findings suggest that migraine patients appeared to be less oriented to this specific coping strategy probably as the expression of migraineur tendency to take completely charge of their daily problems and to feel responsible for all the eventually failures. This coping approach leads the migraine patient to rely exclusively on their own resources and to feel overwhelmed by the surrounding reality, triggering a vicious circuit that could cause the genesis and probably the chronicity of headache.

The present study revealed a negative correlation between “turning to religion” and the impact of migraine on ability to function in the several different situations of migraine patient's life (e.g., the lower the exploitation of the “turning to religion” coping strategy, the higher the HIT-6 score is). These findings support the idea that reduced use of turning to religion might implicate a greater impact on the patient's life.

However, the causal link between migraine and poor turning to religion coping strategy can be hypothesized but not established with certainty and deserves to be better investigated in longitudinal studies.

In the present study, MwoA patients did not show any difference with HCs regarding the perception of stress, partially in disagreement with recent observations, suggesting a close relationship between stress perceived and migraine attacks [30]. This unexpected finding may reflect the idea that migraineurs tend to develop physical symptoms to cope stressful events.

Furthermore, it is noteworthy that, in the present study, MwoA patients did not differ from HCs for psychological symptoms (depression and anxiety), but achieved a significantly lower score on MoCA. This last finding would be in line with the previous study revealing an association between migraine and cognitive defects [31], but we did not observe significant correlations between turning to religion and MoCA scores.

Taken together, these observations could suggest that, independently from levels of anxiety and depression, turning to religion would not be an expression of cognitive functions in migraineurs, differently from what observed in other brain pathologies [32, 33].

Our study had several strengths and limitations. The inclusion of a sample of patients homogeneous for the type of migraine (all MwoA patients) and drug-naïve for preventive pharmacological therapies can be considered as strength of the study, suggesting that reduced use of some adaptive coping strategies can occur in the natural history of MwoA, likely predisposing to the genesis and probably the chronicity of migraine. However, on the other hand, these features may represent a limitation about generalizability of the findings, for example, to MwoA patients taking antimigraine preventive pharmacological therapies. Moreover, the cross-sectional nature of our study did not allow us to ascertain whether abnormal coping strategies can herald clinically relevant worsening and chronicization of migraine. Furthermore, our sample was composed mainly of women, as migraine is strongly related to gender, but this might also limit the generalizability of the results.

Finally, we did not explore the possible relationship between personality traits (e.g., neuroticism) and coping strategies; therefore, this issue deserves to be investigated. Moreover, we did not explore the possible influence of a tendency to complaint on perceived stress score; therefore, future studies should explore this issue.

Our study will not oversimplify the multifaceted migraine pathophysiology suggesting a unique causative role of the abnormal coping strategies, but it could provide a further insight into the complex scenario of migraine mechanisms. Moreover, we believe that the evaluation of coping strategies in migraineurs may be relevant in the clinical routine in order to identify those patients at high risk for frequent migraine attacks. Indeed, interventions to improve spiritual coping strategies [34], self-empowerment, and sense of personal wholeness by unifying the biopsychosocial perspectives [26], restoring an adaptive relationship with the surrounding, and reducing the allostatic load in migraine patients may, in turn, have a positive impact on the migraine burden.

## Figures and Tables

**Table 1:** Demographic and clinical data in patients with migraine without aura and healthy controls.

Parameter	Group	Mean ± SD	*p* value
Gender	MwoA	52 F/9 M	—
	HC	52 F/9 M	
Age (years)	MwoA	33.7 ± 11.1	0.833
	HC	34.1 ± 11.1	
Education (years)	MwoA	12.3 ± 3.4	0.736
	HC	12.1 ± 3.5	
Disease duration (years)	MwoA	14.1 ± 10.9	
Frequency (days/month)	MwoA	5.8 ± 4.4	
MIDAS	MwoA	26.9 ± 20.1	
HIT-6	MwoA	61.1 ± 5.7	
NRS of pain intensity during migraine attacks	MwoA	8.3 ± 0.8	

The values are expressed as mean ± standard deviation. MwoA: migraine without aura; HC: healthy control; MIDAS: Migraine Disability Assessment Scale; HIT-6: Headache Impact Test-6; NRS: numerical rating scale; *F*: Fisher test; MoCA: Montreal Cognitive Assessment; BDI-II: Beck Depression Inventory II; AES: Apathy Evaluation Scale; STAI-Y: Spielberg State and Trait Anxiety Inventory-Form Y; COPE: Coping Orientation to Problems Experienced; PCI: Proactive Coping Inventory; CISS: Coping Inventory for Stressful Situation; PSS: Perceived Stress Scale.

**Table 2:** Neuropsychological, psychological, and coping data in patients with migraine without aura and healthy controls.

	MwoA patients (*n* = 61)	Healthy controls (*n* = 61)	*F*	*p*
*MoCA*	22.21 ± 2.46	25.84 ± 2.01	73.147	<0.001
BDI-II	10 ± 8.25	8.02 ± 6.59	2.149	0.145
AES-S	29.80 ± 5.24	29.23 ± 5.29	0.364	0.548
STAIY-1	40.10 ± 11.28	40.46 ± 12.23	0.029	0.866
STAIY-2	41.64 ± 9.46	40.18 ± 9.57	0.704	0.403
PSS	17.33 ± 6.57	17.87 ± 6.67	0.198	0.657
PCI: proactive	36.74 ± 4.53	36.00 ± 4.53	0.807	0.371
PCI: reflective	31.44 ± 5.35	31.23 ± 4.29	0.059	0.809
PCI: strategic	10.18 ± 2.58	10.57 ± 2.06	0.861	0.355
PCI: preventive	27.16 ± 5.03	26.21 ± 4.92	1.112	0.294
PCI: instrumental	20.64 ± 4.86	21.67 ± 4.73	1.412	0.237
PCI: emotional support	13.98 ± 2.89	14.67 ± 2.92	1.714	0.193
PCI: avoidance	4.98 ± 1.87	5.38 ± 2.16	1.148	0.286
PCI: total	145.30 ± 18.57	145.56 ± 16.26	0.007	0.934
COPE: social support	29.48 ± 7.03	29.59 ± 7.53	0.008	0.931
COPE: denial	23.64 ± 5.38	23.21 ± 4.74	0.215	0.643
COPE: positive reinterpretation	31.61 ± 5.65	30.77 ± 5.93	0.635	0.427
COPE: acceptance	30.05 ± 5.48	29.57 ± 5.74	0.219	0.641
*COPE: turning to religion*	22.08 ± 5.19	24.70 ± 4.44	8.989	0.003
COPE: total	136.85 ± 15.91	137.85 ± 17.85	0.107	0.745
CISS: task solution	49.36 ± 9.43	46.95 ± 10.75	1.730	0.191
CISS: rumination	47.64 ± 11.22	45.80 ± 9.97	0.912	0.341
CISS: aggression	48.31 ± 10.69	49.72 ± 11.67	0.484	0.488
CISS: distraction	48.20 ± 11.31	50.49 ± 11.38	1.247	0.266
CISS: social diversion	48.59 ± 10.25	49.15 ± 11.81	0.077	0.781

The values are expressed as mean ± standard deviation. MwoA: migraine without aura; *F*: Fisher test; MoCA: Montreal Cognitive Assessment; BDI-II: Beck Depression Inventory II; AES: Apathy Evaluation Scale; STAI-Y: Spielberg State and Trait Anxiety Inventory-Form Y; COPE: Coping Orientation to Problems Experienced; PCI: Proactive Coping Inventory; CISS: Coping Inventory for Stressful Situation; PSS: Perceived Stress Scale.

## Data Availability

Clinical, neuropsychological, and statistical data will be available upon request from any qualified investigator.

## Abbreviations

MwoA: Migraine without aura
TTH: Tension-type headache
CM: Chronic migraine
HCs: Healthy controls
COPE: Coping Orientation to Problems Experienced
CISS: Coping Inventory for Stressful Situation
PCI: Proactive Coping Inventory
PSS: Perceived Stress Scale
NRS: Numerical rating scale
MoCA: Montreal Cognitive Assessment
MIDAS: Migraine Disability Assessment Scale
HIT-6: Headache Impact Test-6
BDI-II: Beck Depression Inventory II
STAI-Y-1 and 2: State and Trait Anxiety Inventory 1 and 2
ANOVA: analysis of variance.

## Additional Points

*Highlights*. In the last years, a relationship between recurrence of migraine episodes and maladaptive coping styles has been suggested. Coping represents a personality trait determining individual differences in the way of reacting to stressful life events (including both actions and related emotions). Patients with MwoA show a significantly reduced use of the “turning to religion” approach, an emotion-focused coping strategy. The evaluation of coping strategies in patients with MwoA may be relevant in the clinical routine in order to identify those patients at high risk for frequent migraine attacks. In a therapeutic perspective, the external strategies (such as the “turning to religion”) seem to be more accessible for treatment than internal coping, more difficult to assess and influence.
